# Gunshot Wounds by the Old and New Armaments

**Published:** 1902-07

**Authors:** L. A. La Garde

**Affiliations:** Washington, D. C., Surgeon U. S. Army. Soldiers’ Home, Washington, D. C.


### Gunshot Wounds by the Old and New Armaments.

By MAJOR L. A. LA GARDE, M. D., Washington, D. C.,
Surgeon U. S. Army.

IN SPEAKING of the modern projectile I shall confine my remarks to that of the hand-weapon class, in contra-distinction to that of the artillery arm. Speaking generally, gunshot wounds, when so specified, have reference to the lesions inflicted by the former for the reason that they inflict on an average over 90 per cent. of all wounds in war.

Before we enter upon the effects of the modern bullet into the tissues it may be interesting to discuss its origin and the causes that led to its adoption. At the onset it may be stated that the modern reduced caliber projectile as we know it today as an engine of warfare, was largely the result of accident.

When the modern tactician became possessed with the rapid means of transporting and concentrating troops, he called upon the ballistician to give him a weapon that should fire the greatest number of shots possible in a given space of time. The military weapons of two decades ago were capable of delivering twenty shots per minute in expert hands. A good example of this class is the Springfield rifle, which we discarded in 1893. It fired an elongated bullet of .45 calibers, made of lead hardened by antimony, weighing 500 grains. The explosive conferred upon this missile an initial velocity of 1,301 foot seconds, and the twist in the barrel—one complete turn in twenty-two inches—gave it a velocity of rotation of 800 turns per second at the muzzle. When fired into unseasoned oak against the grain, three feet from the muzzle, it penetrated five and one-half inches.

In casting about for a more effective arm or one having a greater rapidity of fire, the gunmaker was at once confronted with the fact that the soldier was already loaded to the maximum. To burden him with more cartridges was simply out of the question, thus it was that the attempt to reduce the caliber and thereby the weight of the ammunition per round was attempted. That the attempt succeeded is shown by the fact that for the same weight the soldier carries nearly twice as much of the new ammunition as he did of the old.

But how does it happen that this effective little bullet that has helped to revolutionise the subject of gunshot wounds became a creation of accident? As already stated, the gunmaker reduced the caliber in order to multiply the number of rounds per man. This reduction in caliber of the rifle added to the difficulty incident to fouling from the unconsumed residue of the old black powder, a circumstance which led to the use of those nitro-compounds which we know under the name of smokeless powders. These powders afforded a great advantage in that they left little or no residue in the barrel, and further it was found they conferred a greater initial velocity, as the gases which they evolve issue in proportionately larger volume. This increase in velocity, most desirable in itself, added quite a complication. The lead bullet, instead of keeping point on, tumbled, that is, it soon lost its stability in flight and went on end over end, to prevent which a shorter twist was given to the rifling. Instead of one complete turn in twenty-two inches, as described in the old rifle, the twist was shortened to one in ten inches. The lead bullet became refractory under the bidding of such a sharp turn. Instead of following the new twist, it stripped through the barrel without revolving properly, a difficulty which was finally obviated by enclosing the lead with a mantle of harder metal like copper, German silver or hard steel, so it is rather by accident than design that we have the steel-jacketed reduced caliber bullet in use by the nations the world over; a bullet that does not deform on impact against the most resistant structure in the body, that travels at the rate of 2,200 foot seconds, and that describes 2,400 turns per second at the muzzle and which is capable of penetrating nineteen and one-half inches of unseasoned oak when fired into at three feet from the muzzle; it appears further that forty of those projectiles can be delivered per minute from a repeating rifle.

This projectile has done more to influence the outcome in gunshot wounds than any other one thing save antisepsis. It has been most appropriately termed a humane bullet, but mind you, it was not the purpose of the ballistician at the onset to invent a humane bullet. He stumbled along in his search to multiply the shots per man, as I have tried to explain, and after overcoming a number of obstacles, his efforts were rewarded with this effective missile. The humane effects of the bullet came about as though a ruling Providence had hovered over his efforts to guide men, to help lessen the carnage of battle and to make war more endurable. It is not the province of the ballastician to employ considerations of humanity when he fabricates the engines of warfare. His aim is to destroy and not to build up. He does it all in the interest of the state, for the sake of patriotism. To alleviate the suffering of the hero, to make war more humane, to reduce to a minimum the pain, the loss of life and limb is the mission of others. That is the mission of our proud profession, so lofty in its aims for the public welfare, to which the medical department of all armies belongs. It is our special privilege to go to war for the sake of patriotism and the lofty considerations of humanity as well.

When our government contemplated a change in its armament, now ten years ago, I was fortunate in receiving the detail to test the efficiency of the new arm. The work was done by firing into ten cadavers with simulated velocities with the new and the old bullet. As a broad principle it may be stated that the lesion inflicted was, as a rule, proportional, first, to the velocity and sectional area of the bullet, and, second, to the resistance which it encountered on impact.

From the evidence obtained in our experiments we gathered that the claim made in favor of the little bullet was well founded. The experimenters naturally awaited the test of battle with great interest. The first opportunity on a sufficient scale occurred in the Spanish-American war and next the Anglo-Boer war. I will contrast our experimental work with what I observed in Cuba as follows:

Eleven conclusions:

1. The experimental evidence showed the shock impressed upon a member increases with the velocity whether a bone is traversed or not. It is always greater with the leaden projectile.

This diminution in shock was one of the serious objections advanced against the adoption of the small bullet by military men. They feared that one wound would not suffice to throw a man *hors du combat*, and that he might be able to go on fighting regardless of the fact that he had been hit a number of times. Whether this is true of savage tribes, or horses in a cavalry charge, it is not true of our American soldiers. Upon inquiry among the line officers in the Santiago campaign I find that, as a rule, to which there are very few exceptions, men when hit fell back to the rear at once; and I can testify to the fact that scores of them walked back to the hospital at Siboney with wounds that were most trifling in their nature.

2. The explosive effects at very short range are about the same for the two projectiles. They continue, however, up to 350 yards with the smaller bullet and cease at about 200 yards with the leaden bullet.

I only saw one case which approached anything like explosive effects in Cuba. That was the case of a captain of the Rough Riders shot in the lower third of the tibia. The wound of entrance was about the caliber of the Mauser bullet that had inflicted it, and the wound of exit was irregularly round, a half-inch in diameter. There were two smaller wounds near the wound of exit, which were undoubtedly made by spiculæ of bone which had been driven forth, acting as secondary missiles. The area of fracture was about four inches above the ankle; it was marked by a cavity in which many loose fragments of bone lay, none of them measuring more than a half-inch. The wall of the cavity showed bony sand driven into the soft parts.

3. The experimenters found that the smaller frontage of the jacketed bullets causes them to inflict injuries resembling subcutaneous wounds when the soft parts alone are traversed, and that the small wounds of entrance and exit and the narrow track of the missiles were favorable circumstances to rapid healing.

The truth of this statement is borne out by the experience of all surgeons in the Santiago campaign. Flesh wounds healed very kindly and rapidly.

4. The next conclusion of the experimenters refers to hemorrhage. The blood-vessels are seldom torn by the small jacketed bullet, and when wounded they are not closed so easily by coagulation as those severed by leaden projectiles.

Some writers have deduced from this statement that alarming or fatal hemorrhage would be more frequent in future battles. The experiences of the surgeons with the line before Santiago do not confirm these apprehensions. Of the 1,400 wounded, as far as I can learn, not one died of external hemorrhage. The brachial and femoral were tied a few times in the base hospital for diffuse aneurism. One case of wound of the subclavian was operated upon in New York and died after the operation. There were five cases of gangrene from injury to blood-vessels which required amputation.

5. Injuries inflicted outside the zone of explosive effects upon the shafts of the long bones always show less comminution with the small bullet of hard exterior. The fissures are often subperiosteal and the fragments are larger.

This was true of the Mauser bullet wounds in Cuba. It was seldom necessary to open up the wounds for the purpose of taking out loose fragments of bone. In a number of instances there was distinct guttering of the compact substance of long bones without fracture. The mobility in some instances was so slight that it was difficult to make out a complete fracture when from the location of the wounds it was certain that the bone had been traversed.

6. The projectiles of hard exterior lodge less frequently in the tissues than the old leaden bullet.

The experience at Santiago among the wounded of both sides has shown a surprisingly large number of lodged balls. Although I am not prepared to state that the small caliber bullet lodges as often as the old discarded leaden bullet, the frequency with which it did lodge was remarked upon by military surgeons generally.

Dr. W. E. Parker, of New Orleans, an acting assistant surgeon in the base hospital, visited the Spanish hospitals in Santiago after the surrender, and in conversation with the Spanish surgeons he learned that our Krag-Jorgensen bullet had not lodged in their wounded as often as their Mauser bullets had lodged in our men. The explanation for this would seem simple enough. It should be remembered that we were on the aggressive in a region that was practically unknown to our troops, whilst the Spaniards were perfectly familiar with every foot of ground over which we must make the advance. As trained soldiers their officers had carefully studied the range at every point. With this valuable information in their favor they were in a position to commence an effective fire at remote ranges, say at 2,000 yards and more. We could not locate them as soon as they located us, and when we did locate them we had to study the range before we could commence an effective fire. It was while we were locating them and studying the range and gradually advancing that they placed so many balls into our soldiers. When we did commence an effective fire we had reached a point where the remaining velocity of our bullet on impact was sufficient to carry it through the body.

There is another explanation which may be gathered from the difference in the energy of the two bullets at remote ranges. Our bullets being larger and heavier than the Mauser have greater energy at 2,000 yards and will penetrate farther in the remote ranges than theirs. Again, ricochet shots, from the thick underbrush and broken ground, undoubtedly favored a certain percentage of lodgment. Many of the officers attributed the lodgment of projectiles to the use of defective ammunition used by the enemy. This point was so susceptible of proof that I instituted experiments to show the relative penetration of the Mauser and Krag-Jorgensen rifles. The tests were made in large blocks of well-seasoned yellow pine fired into, across the grain, three feet from the muzzle. The penetration of the Krag-Jorgensen ammunition was 24 inches plus, whilst that of the Mauser ammunition exceeded ours by nearly 10 inches, a demonstration which at once set at rest the idea of lodgment from the defective ammunition of the enemy.

7. The old leaden bullet more often leaves fragments of lead in the foyer of fracture.

This is so true that it needs no contradiction. The leaden bullet was so soft that it often separated into a number of fragments upon striking resistant bone, whilst the steel-jacketed bullet seldom encounters resistance enough in the human body to disintegrate it.

8. As the projectiles of smaller caliber are less apt to lodge or to carry foreign substances into the wounds we will expect to find fewer cases of suffering due to the remote effects of unextracted foreign bodies.

This is true of the smaller bullet, as shown in Cuba. There were but few instances where clothing or part of the equipment was carried into the wound.

9. The frontage of the jacketed bullet being much less and the fact that it does not lodge as often as the larger leaden bullet will contribute to increase the percentage of recoveries in gunshot wounds of the lungs.

That was especially true of the wounded in Cuba. As a rule the wounds of the lungs were apparently so trivial that it was difficult to restrain the men in a recumbent posture. The mortality now is but 27 per cent. as compared to 60 per cent. in the Civil War.

10. Beyond the zone of explosive effects the projectiles of hard exterior almost invariably perforate or gutter the joint ends of bones, and the lesions of the articulations are never so grave.

This conclusion tallies exactly with what we saw in Cuba. I do not recall a formal excision of a joint for the mechanical effects of the Mauser bullet. Joints were opened to turn out blood-clots, and in one instance of the knee I particularly remember, to locate a lodged ball, but never for the purpose of performing an excision. There were at least twenty cases of gunshot injury of the knee-joint alone. These were immobilised and shipped north; and as far as I have been able to ascertain they have done well. These results are a great contrast to those inflicted by the larger leaden bullet, which by its highly destructive effects must have caused a number of resections and amputations.

11. The projectiles of hard exterior are more humane than the old; resections and amputations will not be so often required hereafter; soldiers will be more often restored to the state useful members of the community instead of cripples and pensioners, and in point of economy, the new projectile confers a great advantage.

This last conclusion is also in accordance with the experience in Cuba. There were but three primary amputations and not one of them was done for injury by the small bullet. They were all the result of shell injuries. From the foregoing I believe we should conclude that the work of the experimenters agrees with the conditions found in war, and that their work was not done in vain.[1]

Soldiers' Home, Washington, D. C.

1. Read and illustrated with the stereopticon at the 27th annual meeting of the Alumni Association, Buffalo University Medical College, May 2, 1902.

